# Real-World Data on Effectiveness and Safety of First-Line Use of Caplacizumab in Italian Centers for the Treatment of Thrombotic Thrombocytopenic Purpura: The Roscapli Study

**DOI:** 10.3390/jcm13216561

**Published:** 2024-10-31

**Authors:** Luana Fianchi, Matteo Bonanni, Alessandra Borchiellini, Federica Valeri, Gaetano Giuffrida, Stephanie Grasso, Claudio Fozza, Michele Ponta, Giovanni L. Tiscia, Elvira Grandone, Nicola Vianelli, Alessandra Dedola, Teresa Pirozzi, Monica Sacco, Stefano Lancellotti, Raimondo De Cristofaro

**Affiliations:** 1 Hematology Unit, Fondazione Policlinico Universitario Agostino Gemelli—IRCCS, 00168 Rome, Italy; 2 Regional Reference Center for Thrombotic and Haemorrhagic Disorders of Hematology, Division Department of Hematology and Oncology, A.O.U. Città della Salute e della Scienza di Torino, 10126 Torino, Italy; aborchiellini@cittadellasalute.to.it (A.B.);; 3 UOS e Centro di Riferimento Regionale di Malattie Ematologiche Rare, Division of Haematology, A.O.U Policlinico-S. Marco, 95123 Catania, Italy; 4 Department of Clinical and Experimental Medicine, University of Sassari, 07100 Sassari, Italy; 5 Thrombosis and Hemostasis Unit, Fondazione IRCCS “Casa Sollievo della Sofferenza”, S. Giovanni Rotondo, and Unità di Ostetricia e Ginecologia, Università degli Studi di Foggia, 71121 Foggia, Italyelvira.grandone@unifgg.it (E.G.); 6 IRCCS Azienda Ospedaliero, Istituto di Ematologia “Seràgnoli”, Universitaria di Bologna, 40121 Bologna, Italyalessandra.dedola@aosp.bo.it (A.D.); 7 Service of Haemorrhagic and Thrombotic Diseases, Fondazione Policlinico Universitario Agostino Gemelli—IRCCS, 00168 Rome, Italy; 8 Dipartimento di Medicina e Chirurgia Traslazionale, Università Cattolica S. Cuore, 00168 Rome, Italy

**Keywords:** thrombotic thrombocytopenic purpura, von Willebrand factor, caplacizumab, ADAMTS13

## Abstract

**Background/Objectives**: Immune thrombotic thrombocytopenic purpura (iTTP) is a thrombotic microangiopathy caused by the formation of anti-ADAMTS13 antibodies. Caplacizumab is approved for the treatment of acute episodes of iTTP in conjunction with plasma exchange (PEX) and immunosuppression. Real-world data for the use of caplacizumab in Italy have been recently published by a limited number of centers located in the northern and middle regions of the country only. **Methods**: A total of 38 patients with iTTP were enrolled in the study in six Italian centers spread over the entire territory of the country. The patients’ data were registered in eCRF. **Results**: All patients achieved normalization of platelet count (median 2.0 days, IQR: 2–4), within a time significantly shorter than in the absence of caplacizumab, as previously reported in other studies. As to the secondary aims, patients treated with caplacizumab had a few exacerbations (4/38 (10.5%)) and relapses (2/38, 5.3%). No deaths or refractoriness were observed in these patients. The total length of hospitalization was 12 days (IQR: 9–18) and only one patient required 2 days of stay in the intensive care unit. Interestingly, when caplacizumab was initiated within the first 3 days, the plasma exchange (PEX) duration was 9 days (IQR: 8–10), which was significantly lower than those reported in previous studies conducted in the absence of caplacizumab. No severe adverse event was described in the caplacizumab-treated patients. **Conclusions**: Caplacizumab reduced exacerbations and refractoriness compared with previously reported standard-of-care regimens. When administered in association with PEX and immunosuppressive therapy, caplacizumab provided rapid normalization of platelet count, which was responsible for lower overall hospitalization time, ICU stay, lower exacerbations and relapses compared to previously reported outcomes of studies carried out without caplacizumab.

## 1. Introduction

Immune thrombotic thrombocytopenic purpura (iTTP) is a thrombotic microangiopathic disorder resulting from the formation of anti-ADAMTS13 antibodies [1]. Caplacizumab is approved in Italy by the regulatory authority (AIFA) for adults with acute episodes of iTTP together with plasma exchange (PEX) and immunosuppression, the latter being considered the standard of care for this disease [1,2]. Several clinical trials assessed caplacizumab as an effective drug for treating TTP patients [3,4]. These positive results have been successively reaffirmed in real-world trials [5,6,7,8,9,10,11]. Even in the pediatric population, the efficacy and safety of caplacizumab have been demonstrated and have been associated with a reduction in PEX use compared with the pre-caplacizumab era [12]. The use of caplacizumab has been shown to be so effective for ameliorating the outcome of TTP patients that some episodes of severe forms of iTTP were successfully treated without plasma exchange [13,14,15]. Based on these findings, we investigated real-world data on the use of caplacizumab in some Italian hospitals scattered across the northern, middle and southern regions of the country and involved in the treatment of TTP patients. This study is a multicenter, retrospective, observational study in patients with immune thrombotic thrombocytopenic purpura (iTTP) treated in six Italian centers with plasma exchange (PEX) and immunosuppressive drugs as a standard of care in association with first-line use of caplacizumab during the period Q4_2019-Q4_2023 (ROSCAPLI, ClinicalTrials.gov ID: NCT05262881). The primary objective of this study is the description and quantification of clinical response in terms of the time to normalization of platelet count in iTTP patients treated with caplacizumab, in addition to PEX and immunosuppression. The secondary objectives include (a) the number of exacerbations, defined as recurrent thrombocytopenia within 30 days after the end of therapy; (b) rate of relapse, defined as a TTP event occurring more than 30 days after the end of daily plasma exchange; (c) refractoriness, defined by the lack of a doubling of platelet count after 4 days of treatment and a lactate dehydrogenase level that remained above the upper limit of the normal range; (d) TTP-related mortality; and (e) evaluation of adverse events.

## 2. Materials and Methods

Demographic, clinical, and analytical data were collected retrospectively from medical records. The protocol of the study was approved by the Institutional Review Board of the Fondazione Policlinico Universitario Gemelli IRCCS (prot. n. 4305/2021). All clinical centers (Torino, Bologna, Roma, Foggia, Catania, Sassari, listed in the Supplementary Materials) reported patient data in an electronic CRF. All patients were diagnosed at their first iTTP episode. At diagnosis and at the follow-up visits, the retrospective study collected information on the following attributes: sex, blood pressure, platelet count, Hb level, white blood cell count, creatinine, schistocytes count, LDH, Coombs assay at diagnosis only, alanine-leucine-aminotransferase (ALT), total and direct bilirubin, troponin above the upper limit of normal (ULN), ADAMTS13 activity, and anti-ADAMTS13 antibodies.

The initiation day of caplacizumab treatment was defined as the day PEX began. Clinical response, exacerbation, and relapse were defined according to criteria previously defined by the International Working Group for Thrombotic Thrombocytopenic Purpura [16,17]. Refractoriness was defined as platelet counts <50 × 10^9^/L and persistently increased lactate dehydrogenase (LDH) levels (>1.5 ULN) despite five PEX, according to the consensus document on the standardization of terminology in thrombotic thrombocytopenic purpura and related thrombotic microangiopathies [17]. The time to normalization of platelet count was defined as the time from the first PEX to the first day with a count of 150 × 10^9^/L. Exacerbation was defined as an unjustified decrease in platelet counts and elevation of LDH within 30 days of the suspension of treatment for clinical remission that required the re-initiation of PEX. The survival analysis was measured in days or months and was defined as the period between the PEX and the occurrence of the event of interest. ADAMTS13 levels during follow-up were measured almost only in patients treated with caplacizumab according to the managed access program requirements. Adverse events were also recorded and graded according to the Common Toxicity Criteria for Adverse Events (CTCAE) v5.0. First PEX to exacerbation was defined as an unjustified decrease in platelet counts and increase in LDH less than 30 days after treatment suspension due to clinical remission and requiring the re-initiation of PEX. 

### Statistical Analysis

Given the exploratory nature of this study, no sample size calculation and no adjustments for multiple testing were performed. All results are given as median and IQR or mean with a 95% confidence interval (CI), and the standard errors of the means, when appropriate, are reported. Statistical analysis was conducted using SPSS (Microsoft, v. 21) and Prism version 8.0.1 (GraphPad Software, San Diego, CA, USA). Figures and tables were designed and composed using SPSS and Microsoft Office (Microsoft, Seattle, WA, USA).

## 3. Results

**Figure 1:**
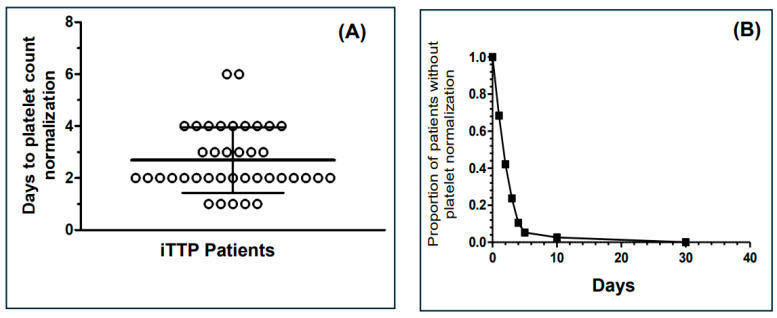
(**A**) Distribution of time to normalization of the platelet count (>150,000/μL) after starting caplacizumab treatment (days). The data concerning longer periods of caplacizumab treatment (>6 days) are censored. The horizontal bars represent the median and IQR values. (**B**) Proportion of patients without platelet normalization as a function of days of caplacizumab treatment.

Thirty-eight patients were enrolled in the study from six Italian centers (Roma, Bologna, Catania, Sassari, Torino, Foggia) that treat iTTP patients. The baseline characteristics of these patients are listed in Table 1. The median PLASMIC score was 5 (IQR: 4–6), suggesting the usefulness of this parameter for promptly treating TTP patients at least with PEX and immunosuppressive agents in centers where ADAMTS13 is not promptly available. On this basis, all patients were indeed treated with PEX and prednisone with 1–1.5 mg/kg per day or the equivalent dose of other corticosteroids (e.g., methylprednisolone, standard therapy). In four patients, PEX was continued two days after the normalization of the platelet count because they showed a difficult normalization of ADAMTS13 level due to the presence of low but significant levels of anti-ADAMTS13 antibodies (0.9–2 U/mL). Six patients (16%) also received rituximab as a first-line immunosuppressive drug. Interestingly, the reason for choosing rituximab use was not correlated with platelet count (median platelet count: 17,000/μL (IQR: 11,500–22,500) vs. 15,500/μL (IQR: 9000–29,250), *p* = 0.865, nor with ADAMTS-13 inhibitors’ titer (median level 22 U/mL (IQR: 9.8–43.4) vs. 25.2 U/mL (IQR: 13.4–82.3), in patients treated and not treated with rituximab, respectively. The reason for such a choice mostly stemmed from clinicians’ personal decisions. The primary endpoint, the median time (days) to normalization of the platelet count (>150,000/μL) after starting caplacizumab treatment, was equal to 2.0 (IQR: 2–4, see Figure 1), in agreement with the results obtained in the Titan and Hercules trials [3,4].

**Figure 2:**
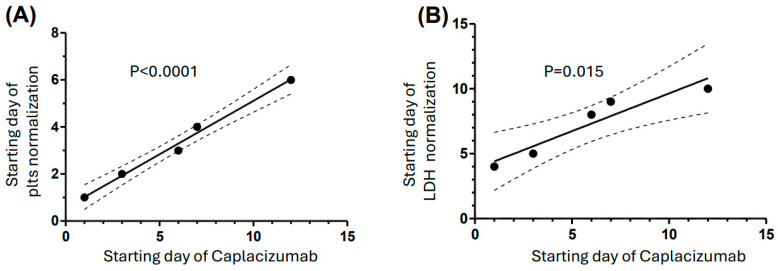
(**A**) Dependence of first day of normalization of the platelet count as a function of the starting day of caplacizumab therapy (slope of the linear regression: 0.45 ± 0.03). (**B**) Dependence of first day of normalization of LDH level as a function of the starting day of caplacizumab therapy (slope of the linear regression: 0.58 ± 0.12). The dotted lines express the 95% confidence intervals.

The starting day of caplacizumab was variable (median time from PEX was 6 days; IQR, 3–12 days), with a median duration of treatment of 33 days (IQR, 31–42 days). No patient received further administration of caplacizumab after 49 days. Caplacizumab was discontinued when the ADAMTS13 activity level was measured at >20 IU/dL, or 30 days after finishing daily plasma exchange therapy. The value of six days is quite high and was largely justified by the date of caplacizumab’s approval by the Italian Medicines Agency (AIFA authorization: January 2020) which had to be further authorized by the regional regulatory institutions (in some cases for the completion of the “compassionate use” procedure during Q4-2019), which took 7–14 days to be activated. Notably, we observed a linear relationship between the temporal interval between the PEX and caplacizumab initiattion and the time of both platelet and LDH normalization (Figure 2): the longer the interval, the longer the time to normalize these parameters. Any 2-day delay in starting caplacizumab therapy causes a 1-day delay in platelet count normalization and a 1.2-day delay in LDH normalization (Figure 2). Furthermore, no significant difference in the delay of caplacizumab initiation since the PEX starting day was found among the clinical centers located in the various regions of Italy (north, center, and south). The median follow-up of the patients was 246 days (IQR: 130–457 days). An important secondary outcome is the financial burden of patient hospitalization when caplacizumab was used for treatment. In these patients, the number of days in which PEX was used was 9 (IQR: 8–10), significantly lower than that observed in previous real-world observational studies conducted in Spain (median: 14, IQR: 7–21.5) [18] and other countries [19] (Table 2). Likewise, only one patient (2.63%) needed to enter an ICU for two days, even after receiving early treatment with caplacizumab. This finding is in line with previous results showing no difference in the length of intensive care unit stay between patients treated and not treated with caplacizumab (Table 2) [18]. Four exacerbation episodes were recorded (10.5%), mostly involving the patients with the initial most severe thrombocytopenia forms (<15,000 platelets/μL). This result is in line with the findings of a recently published meta-analysis [20]. Likewise, the relapses were observed in two patients, and in one case the patient developed gastrointestinal cancer ≅450 days after the initial TTP diagnosis and treatment. Notably, no refractoriness was observed, and this finding most likely contributed to the absence of mortality among this patient cohort. No correlation was found between the ADAMTS13 inhibitors’ titer and the development of ischemic stroke, which occurred in three patients (*p* [two-tailed] = 1.00). Notably, in one of these three patients with an ADAMTS13 activity <1 IU/dl, inhibitor titer of 62.3 U/mL and 17,000 plt/μL, an acute myocardial infarction was also observed (hs-cTnT: 10,123 ng/L). This finding would suggest that in most severe TTP cases, both the measurement of biomarkers of myocardial necrosis and neuroimaging studies should always be performed, as proposed in previous studies to avoid longer-term morbidities affecting these patients [21,22]. No venous thromboembolism was observed in this patient cohort. Concerning the immunosuppressive therapy, the patients were treated with high-dose corticosteroids (mostly prednisone, 1–1.5 mg/Kg b.w.). As anticipated above, six patients were also treated with rituximab (at the usual dose of 375 mg/m2 once a week for four weeks).

As to safety, the clinicians did not report any adverse effect in patients treated with caplacizumab, excluding minor local irritative skin phenomena at the drug injection site and, in two patients, a metrorrhagia episode that did not require medical intervention. In the six centers participating in this study, both ADAMTS13 activity and inhibitors were measured in 36 out of 38 patients (94.7%), representing an acceptable diffusion and utilization in Italian clinical laboratories of this test that facilitates the differential diagnosis of TTP compared with other forms of thrombotic microangiopathies. 

## 4. Discussion

This observational, retrospective, multicenter study, conducted in six clinical centers in Italy on 38 TTP patients, scattered in different geographic areas of the country, showed that the efficacy and safety of caplacizumab were similar to those reported in previous real-world studies conducted in different European countries [7,17]. Namely, normalization of the platelet count occurred within less than 3 days, and this result contributed to eliminating mortality and significantly reducing the exacerbation rate reported in various epidemiological studies carried out in Canada, France, and Spain in the pre- and post-caplacizumab era, respectively [2,5,18]. In the Canadian trial carried out over the late 1980s and early 1990s, in which about 100 patients with iTTP were randomly assigned to receive either PEX or fresh-frozen plasma infusion, platelet count normalization was mainly attained in the PEX group (51 patients) after a mean of 15.8 days (range: 3 to 36) [2]. In the French study, the patient cohort treated with caplacizumab recovered durable platelet count ~2 times more rapidly than those of the historical cohort (5, IQR:4-6; vs. 12, IQR: 6-17 days; *p* < 0.01) [5]. In the Spanish observational trial, where adult iTTP patients were treated with caplacizumab in association with plasma exchange (PEX) and immunosuppression, the median time to platelet count normalization was shorter in patients not treated with caplacizumab: 8.5 days (IQR, 6–12.5) vs. 14 (IQR, 7–21) days (*p* = 0.009). Likewise, in the Milan TTP registry, the median time to first normalization of platelet count after caplacizumab start was equal to 4 (IQR: 3–4) [10]. In historical cohorts, exacerbations (typically referring to the recurrence of TTP symptoms within 30 days after achieving an initial remission with PEX) were reported in up to 40% of patients [2,11,18]. In our study, only 2 patients out of 38 (5.3%) experienced relapses. In the pre-caplacizumab era, this phenomenon occurred in about 20–50% of patients over a longer-term follow-up [10,11,19,23]. Altogether, these results also have implicit repercussions on the economic burden for the management of TTP patients, especially when the subjects experience exacerbation and relapse of the disease that causes long-term cardiovascular and neurological complications [21,24,25,26]. Accordingly, a pharmacoeconomic analysis of caplacizumab use in iTTP patients showed that caplacizumab is associated with PEX and immunosuppression is cost-effective, allowing patients to have a good quality of life and the hospital to achieve greater efficiency in managing the burden of this life-threatening disease [26]. As to the immunosuppressive treatment, the use of rituximab in this patient cohort (16%) seems to only partially follow the recommendations provided by previous expert opinions and recent ISTH guidelines that provided evidence that the benefits of rituximab in a large proportion of cases exceed the risks and are much stronger, especially for preventing relapse and refractoriness of the disease [27,28,29]. The present findings strongly suggest that caplacizumab should be used in the first instance, as it lowers iTTP-related mortality and refractoriness and decreases the number of daily PEX and hospital and ICU stays. However, for the sake of truth, iTTP-related mortality has been decreasing irrespective of caplacizumab use over the last few decades, and several observational studies reported mortality rates between <1% and 20% [6,19,30,31,32]. The improvement of diagnostic tools and therapeutic management of iTTP patients has contributed to the progressive reduction in iTTP-related mortality. Hence, the limited number of mortality episodes in caplacizumab-treated patients should be interpreted with caution. We remark that in a recent study concerning the impact of caplacizumab on treatment outcomes in iTTP patients, an NNT = 35 is sufficient to observe a 2.87% reduction in mortality [33]. Our data confirm this finding, strongly suggesting that caplacizumab can significantly reduce mortality as well as refractoriness of the disease. Another interesting aspect emerging from this study concerns the relationship between the delay of caplacizumab treatment and the time of both platelet and LDH normalization. Our data showed that any 2-day delay in starting caplacizumab therapy causes a 1-day delay in platelet count normalization and a 1.2-day delay in LDH normalization (Figure 2). Thus, any delay in caplacizumab use can negatively influence both the short-term and long-term negative effects on the cardiovascular, hematological, and neurological sequelae of the disease. As an ancillary notation, no significant difference in the availability and timing of caplacizumab initiation since the PEX starting day was found among the clinical centers located in the various regions of Italy (north, center, and south), and this reveals a uniform response from the regulatory institutions at a regional level. There are limitations to our study, including the study’s retrospective design, small sample size, and possible bias in data collection. Heterogeneity in treatment regimens resulted from patients receiving care in different centers. The comparability of data with those from RCTs is limited by the varying times at which caplacizumab was administered following the starting episode.

In conclusion, the first-line use of caplacizumab in addition to PEX and immunosuppressive treatment in iTTP patients showed clear positive effects on the clinical outcomes of the disease, significantly reducing the number of days for the normalization of platelet count, exacerbations, and relapse episodes. Accordingly, a recent study showed that the use of caplacizumab, even without PEX, can provide good results for the management of iTTP patients [15]. Although further clinical studies will have to be conducted to validate the mortality reduction induced by caplacizumab, the above-mentioned effects could contribute to significantly reducing the long-term cardiovascular and neuropsychiatric complications that severely affect the health and quality of life of patients previously affected by iTTP. 

## Figures and Tables

**Table 1:** Median and IQR values of baseline laboratory parameters of iTTP patients at diagnosis (n = 38) *.

Gender (F:M, %)	Age (yr)	BMI	Hb (gr/dL)	Plt (×10^9^/L)	WBC (×10^9^/L)	LDH (U/L)	Total Bilirubin (mg/dL)	hsTnI (pg/mL)	AD13 ^§^ (U/dL)	AD13 ° Inhibitor titer (U/dL)	ALT (U/L)
(78.9:21.1)	43 (39–56)	25.4 (20.7–29.9)	8.75 (7.1–16.20)	17 (9–28)	8.89 (5.96–10.64)	634 (320–1064)	1.9 (1.4–3.15)	224 (103–312)	0.005 (0–0.4)	24.5 (14–67.8)	20 (12.5–27)

* All values represent the median along with IQR in parentheses. ^§^ ADAMTS13 activity was measured in 36 out of 38 patients. ° Assessed as BU/mL. Schistocytes were present (26/38, 68.4%) at a median of 2.2% (IQR: 1.9–4.2%). The median PLASMIC score was 5 (IQR: 4.0–6.3). The Glasgow score was in all cases equal to 15 (minor brain injury), except two cases with values equal to 3 and 4, respectively. In these cases, no eye movement, best motor and verbal response were present in the absence of a typical ischemic stroke. No patient had renal insufficiency (always eGFR > 60 mL/min).

**Table 2:** Secondary outcomes in 38 TTP patients treated with caplacizumab.

Parameter	Result (Number of Cases or Days, and % or IQR)
Death	0 (0%)
TTP relapse	2 (5.3%)
Serious Adverse Events (SAEs)	0 (0%)
Exacerbations	4 (10.5%)
Refractoriness	0 (0%)
Number of PEX days (median, IQR)	9 (8–10)
Days of stay in ICU	2 days for 1 patient
Days of hospitalization (median, IQR)	12 (9–18)
Safety assessments, as reported descriptively by the clinicians at each center	Nothing to report

## Data Availability

The datasets generated during and/or analyzed during the current study are available from the corresponding author upon reasonable request.

## Supplementary Materials

The following supporting information can be downloaded at https://www.mdpi.com/article/10.3390/jcm13216561/s1, Figure S1 Geographical localization of clinical centers with the relative P.I. names participating in the ROSCAPLI study.
