# Supplementary material for: Real-World Data on Effectiveness and Safety of First-Line Use of Caplacizumab in Italian Centers for the Treatment of Thrombotic Thrombocytopenic Purpura: The Roscapli Study
Source: J Clin Med. 2024 Oct 31;13(21):6561. doi: 10.3390/jcm13216561 (PMC11546578; doi:10.3390/jcm13216561)
Supplement: Supplementary file 1 [file jcm-13-06561-s001.zip › jcm-3199023-supplementary.pdf]

Supplementary Materials

REAL WORLD DATA ON EFFECTIVENESS AND SAFETY OF FIRST LINE USE OF CAPLACIZUMAB IN ITALIAN CENTERS FOR THE TREATMENT OF THROMBOTIC THROMBOCYTOPENIC PURPURA: THE ROSCAPLI STUDY

Luana Fianchi[1], Matteo Bonanni[1], Alessandra Borchiellini[2], Federica Valeri[2], Gaetano Giuffrida[3], Stephanie Grasso[3], Claudio Fozza[4], Michele Ponta[4], Giovanni L. Tiscia[5], Elvira Grandone[5], Nicola Vianelli[6], Alessandra Dedola[6], Teresa Pirozzi[7], Monica Sacco[8], Stefano Lncellotti[7], and Raimondo De Cristofaro[7,8]

[1]Hematology Unit, Fondazione Policlinico Universitario Agostino Gemelli - IRCCS, Rome, Italy, [2] Regional Reference Center for Thrombotic and Haemorrhagic Disorders of Hematology, Division Department of Hematology and Oncology, A.O.U. Città della Salute e della Scienza di Torino, Torino, Italy; [3] UOS e centro di riferimento regionale di Malattie Ematologiche Rare, Division of Haematology, A.O.U Policlinico-S. Marco, Catania, Italy; [4] Department of Clinical and Experimental Medicine, University of Sassari, Sassari, Italy; [5] Thrombosis and Hemostasis Unit, Fondazione IRCCS "Casa Sollievo della Sofferenza", S. Giovanni Rotondo, and Unità di Ostetricia e Ginecologia, Università degli Studi di Foggia, Foggia, Italy; [6] IRCCS Azienda Ospedaliero-Universitaria di Bologna, Istituto di Ematologia "Seràgnoli", Bologna, Italy;; [7]Fondazione Policlinico Universitario Agostino Gemelli - IRCCS, Rome, Italy; [8] Dipartimento di Medicina e Chirurgia Traslazionale, Università Cattolica S. Cuore, Roma, Italy.

Figure S1

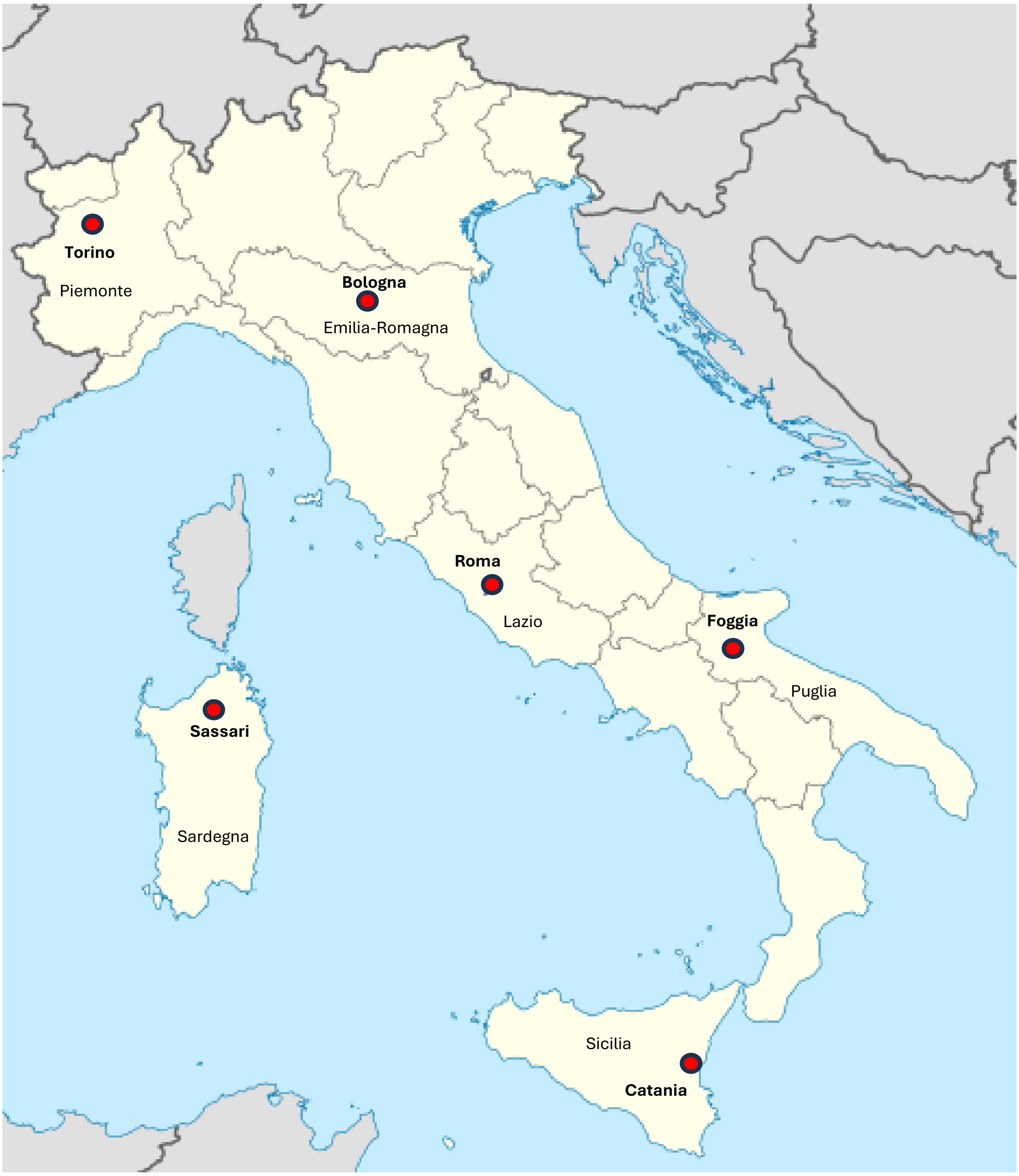
Torino
Piemonte
Bologna
Emilia-Romagna
Roma
Lazio
Foggia
Puglia
Sassari
Sardegna
Sicilia
Catania

- **P.I. Dr. Luana Fianchi**
  Dr. Matteo Bonanni
  Fondazione Policlinico Universitario Agostino Gemelli, Largo Gemelli, 8 -ROMA-ITALY Dipartimento di Scienze di laboratorio ed ematologiche
  Università Cattolica S. Cuore
  e-mail: luana.fianchi@policlinicogemelli.it – 17 patients

- **P.I. Dr. Alessandra Borchiellini**
  Dr. Federica Valeri
  A.O.U. Città della Salute e della Scienza di Torino, ITALY
  e-mail: aborchiellini@cittadellasalute.to.it – 6 patients

- **P.I. Dr. Gaetano Giuffrida**
  Dr. Stephanie Grasso
  Ematologia, Policlinico Rodolico - San Marco, Catania, Italy
  e-mail: gaegiuffrida@gmail.com – 6 patients

- **P.I. Prof. Claudio Fozza**
  Dr. Michele Ponta
  Ssvcienze Mediche Chirurgiche e Sperimentali
  Facoltà di MEDICINA, CHIRURGIA E FARMACIA
  Università di Sassari -Viale San Pietro, 43 - 07100 SASSARI, ITALY
  e-mail: cfozza@uniss.it - 4 patients

- **P.I. Dr. Giovanni Tiscia**
  Prof. Elvira Grandone
  Fondazione IRCCS Casa Sollievo della Sofferenza, Foggia
  e-mail: g.tiscia@operapadrepio.it – 3patients

- **P.I. Nicola Vianelli**
  Dr. Alessandra Dedola
  AOU Bologna, Policlinico Sant'Orsola
  e-mail: nicola.vianelli@aosp.bo.it ; nicola.vianelli@unibo.it – 3 patients

Figure S1. Geographical localization of clinical centers with the relative P.I. names participating in the ROSCAPLI study.
